# Traditional Oral Hygiene Practices and Their Effectiveness: A Systematic Review of the Evidence

**DOI:** 10.3290/j.ohpd.c_2475

**Published:** 2026-02-11

**Authors:** Muhammad Shahzad, Habab Ali Ahmad, Sriram Ambadi, Shenita Peterson, Irene Yang

**Affiliations:** a Muhammad Shahzad Associate Professor, Faculty of Dentistry, Zarqa University, Zarqa, Jordan. Conceptualization, methodology, wrote original draft, read and approved the final version of the manuscript.; b Habab Ali Ahmad Lab Instructor, Department of Biological and Health Sciences, Pak-Austria Fachhochschule Institute of Applied Sciences and Technology, Khyber Pakhtunkhwa, Pakistan. Methodology, reviewed and edited the manuscript.; c Sriram Ambadi Undergraduate Student, Emory College of Arts & Sciences, Emory University, Atlanta, Georgia, USA. Methodology, reviewed and edited the manuscript. Current affiliation: Medical Student, School of Medicine, University of California San Diego, San Diego, CA, USA.; d Shenita Peterson Systemic Review Coordinator and Public Health Informationist, Woodruff Health Science Center Library, Emory University, Atlanta, Georgia, USA. Methodology, reviewed and edited the manuscript.; e Irene Yang Assistant Professor, Nell Hodgson Woodruff School of Nursing, Emory University, Atlanta, Georgia, USA. Conceptualization, methodology, reviewed and edited the manuscript, funding acquisition.

**Keywords:** alternative oral care, chewing stick, herbal dentifrices, miswak, oil pulling.

## Abstract

**Purpose:**

Given the increasing interest in natural and sustainable oral hygiene options, this systematic review evaluates the efficacy of traditional oral hygiene practices, specifically miswak and oil pulling, in maintaining oral health compared with conventional practices, addressing the question: how do these practices affect plaque and gingival outcomes relative to toothbrushing and standard rinses? We hypothesized non-inferiority for short-term plaque/gingivitis control, with limited evidence for longer-term outcomes.

**Materials and Methods:**

A systematic review was conducted following PRISMA guidelines. Comprehensive literature searches were performed in PubMed, Embase.com, Global Health (CABI), Scopus, and Web of Science up to April 2024 using predefined inclusion criteria, focusing on randomized controlled trials and quasi-experimental designs that evaluated traditional oral hygiene methods against conventional practices. Data extraction, quality assessment, and risk bias analysis were conducted independently by two reviewers. Preliminary pilot searches (targeted scoping) were undertaken to refine concepts, eligibility criteria, and keywords. No formal scoping review was registered.

**Results:**

Thirty-one studies met the inclusion criteria. Miswak statistically significantly reduced plaque accumulation and gingival inflammation, performing comparably to or better than conventional toothbrushes. Herbal dentifrices and mouthwashes containing neem, clove, turmeric, and ginger exhibited antimicrobial properties and were as effective as fluoridated products in reducing plaque and gingival inflammation. Oil pulling with sesame or coconut oil showed moderate reductions in microbial load and improved gingival health, though findings varied compared to chlorhexidine mouthwash. Indigenous methods, (e.g., finger brushing, twigs, charcoal) offered accessibility and sustainability benefits but lacked extensive clinical validation.

**Conclusion:**

Traditional oral hygiene practices may offer benefits similar to conventional methods. Nonetheless, methodological limitations and variability among studies necessitate cautious interpretation of these findings. Further thorough research is required to confirm their efficacy and integration into modern oral care.

Despite the rapid developments in dental care technologies and preventive measures, the global burden of oral diseases remains a significant public health challenge.^15,28^ Recent estimates suggest that oral diseases affect over 2.3 billion people across the globe. These oral diseases mainly involve dental caries (tooth decay) caused by accumulation of plaque and demineralization by acid-producing bacteria and periodontal disease, which affects the gums and the supporting structures of the teeth.^14^ Untreated periodontal disease has also been linked to systemic health conditions such as cardiovascular disease and diabetes.^9,22^ Both highly prevalent oral diseases are preventable. Effective oral hygiene practices are, therefore, essential for both oral and systemic health.^3^

While conventional oral hygiene practices, such as the use of fluoridated toothpaste and regular dental visits, have been effective in mitigating these conditions in developed nations, large populations in resource-limited settings lack access to such preventive care.^39^ As a result, there is increasing interest in exploring alternative and traditional oral hygiene practices, which have been employed for centuries across various cultures.^29^ This increasing interest in alternative and traditional oral hygiene practices presents an opportunity to address gaps in oral health care and potentially uncover novel substances that may be effective in preventing and controlling oral diseases.

Traditional or alternative oral hygiene practices involve culturally specific and indigenous methods for oral care. These methods have been used across the world for centuries and potentially offer a viable, cost-effective solution for maintaining oral health.^38^ For example, the Ayurvedic tradition in India emphasizes the use of herbal products for oral hygiene and care. A number of plant-based products and remedies such as neem, turmeric, betel leaves and clove oil, have been used for centuries and across generations due to their antimicrobial properties.^5,34^ Similarly, a number of different plant extracts throughout Africa have been shown to reduce oral pathogens and promote oral health.^24^ Miswak (siwak, meswak) refers to a natural chewing stick, commonly from *Salvadora persica*. Users trim/soften the tip, chew lightly until fibers splay into a brush, and clean teeth with short vertical/circular strokes, refreshing the tip frequently. Oil pulling (Ayurvedic kavala/gandusha), on the other hand, involves swishing edible oil (typically sesame or coconut) throughout the mouth and between teeth, then expectorating; customary protocols specify continuous swishing for approximately 5 to 20 minutes before routine toothbrushing. The use of miswak, particularly in the Middle East and North Africa, holds not only cultural but also religious significance, particularly in Islamic traditions.^25^ These traditions have developed alongside local ecology, relying on available resources and have been passed down through generations as valuable practices for maintaining oral health and hygiene. There is a growing interest in the developed world in exploring alternative methods for oral hygiene and care. The trend is primarily driven by concerns over the chemical content of commercial oral care products, environmental sustainability of the traditional products, and a preference for more “natural” options.^57^ This trend has led to a resurgence in interest in traditional practices and ingredients. For example, the increasing preference for natural and sustainable products in developed nations provides an opportunity to integrate traditional methods into modern oral care strategies, including the increasing popularity of herbal-based toothpastes and oral rinses.

Despite the wide use of traditional oral hygiene practices across different cultures and religion, many lack comprehensive scientific validation. For example, while miswak and neem twigs have been studied for their antimicrobial properties, the literature remains fragmented regarding their effectiveness for clinical outcomes, with few systematic reviews assessing their long-term efficacy compared to conventional methods.^29^ Comparative studies between traditional and modern approaches are needed to determine the effectiveness of traditional practices in preventing oral diseases and reducing microbial loads. While some research highlights the potential benefits of these practices, such as the antimicrobial effects of herbal remedies, further studies are required to explore their mechanisms of action and validate their use in modern preventive strategies.^10^ The current systematic review, therefore, seeks to answer the question: “how effective are traditional oral hygiene practices (intervention) in preventing oral diseases and improving oral health (outcomes) compared with conventional toothbrushing and standard mouthrinses (comparators)?”. To address this, the review aims to summarize and critically evaluate research studies reporting the efficacy of various alternative and traditional oral hygiene practices in maintaining oral health and treatment or prevention of oral diseases. We sought to identify areas where traditional methods have demonstrated promise, while also highlighting gaps in the literature that warrant further research. Ultimately, this review will contribute to a better understanding of how traditional practices can be integrated into modern oral hygiene strategies, particularly in resource-limited settings.

## MATERIALS AND METHODS

The Preferred Reporting Items for Systematic Reviews and Meta-Analyses (PRISMA) statement (2020) was used as the reporting guideline. No formal scoping review was conducted; instead, we performed targeted pilot searches to map terms, refine eligibility, and finalize the strategy prior to full screening.

### Search Methodology

In collaboration with an information specialist, a comprehensive search strategy was developed in PubMed using an iterative process in the spring of 2024. The research question was broken down into the following concepts: “oral health behaviors,” “traditional or alternative” and “oral health materials.” Keywords and controlled vocabulary were identified for each concept by the research team, and the approved search was executed in April 2024. The search was translated and executed in the following bibliographic databases: Embase.com, Global Health (CABI), Scopus (Elsevier), and Web of Science (Clarivate) which is comprised of the following indices: Science Citation Index Expanded (SCIE), Social Sciences Citation Index (SSCI), Arts & Humanities Citation Index (AHCI), Emerging Sources Citation Index (ESCI), Conference Proceedings Citation Index (CPCI), Book Citation Index (BKCI), Current Chemical Reactions, Index Chemicus (see supplementary Table 1). Each search was limited to English, humans, and primary articles. The search result records were imported into EndNote 21 for data management. One thousand six hundred ninety-eight (1698) records were imported into the web-based application Covidence and, after de-duplication, 961 records were screened for inclusion.

**Table 1:** Inclusion and exclusion criteria used to determine study eligibility

Category	Inclusion criteria	Exclusion criteria
Population	Humans and animals, no exclusions based on age, sex, or region	Cancer patients
Intervention	Traditional/cultural oral hygiene practices (e.g., Miswak, chewing stick, oil pulling, charcoal, dandasa, oak bark); herbal/natural oral hygiene products; traditional medicines	Toothbrushing, flossing, mouthwashing, Waterpik; interventions addressing oral symptoms
Comparator	Conventional oral hygiene methods (e.g., toothbrushing, flossing, mouthwashing)	Not applicable
Outcomes	Oral health clinical outcomes (e.g., caries, periodontal health, whitening, oral symptoms); oral microbiome	Non-oral clinical outcomes; cancer-related outcomes
Study design	Randomized controlled trials (RCTs), quasi-experimental studies	Qualitative research, systematic reviews, commentaries/opinions/editorials, descriptive studies, observational studies, case reports


### Screening

**Fig 1:**
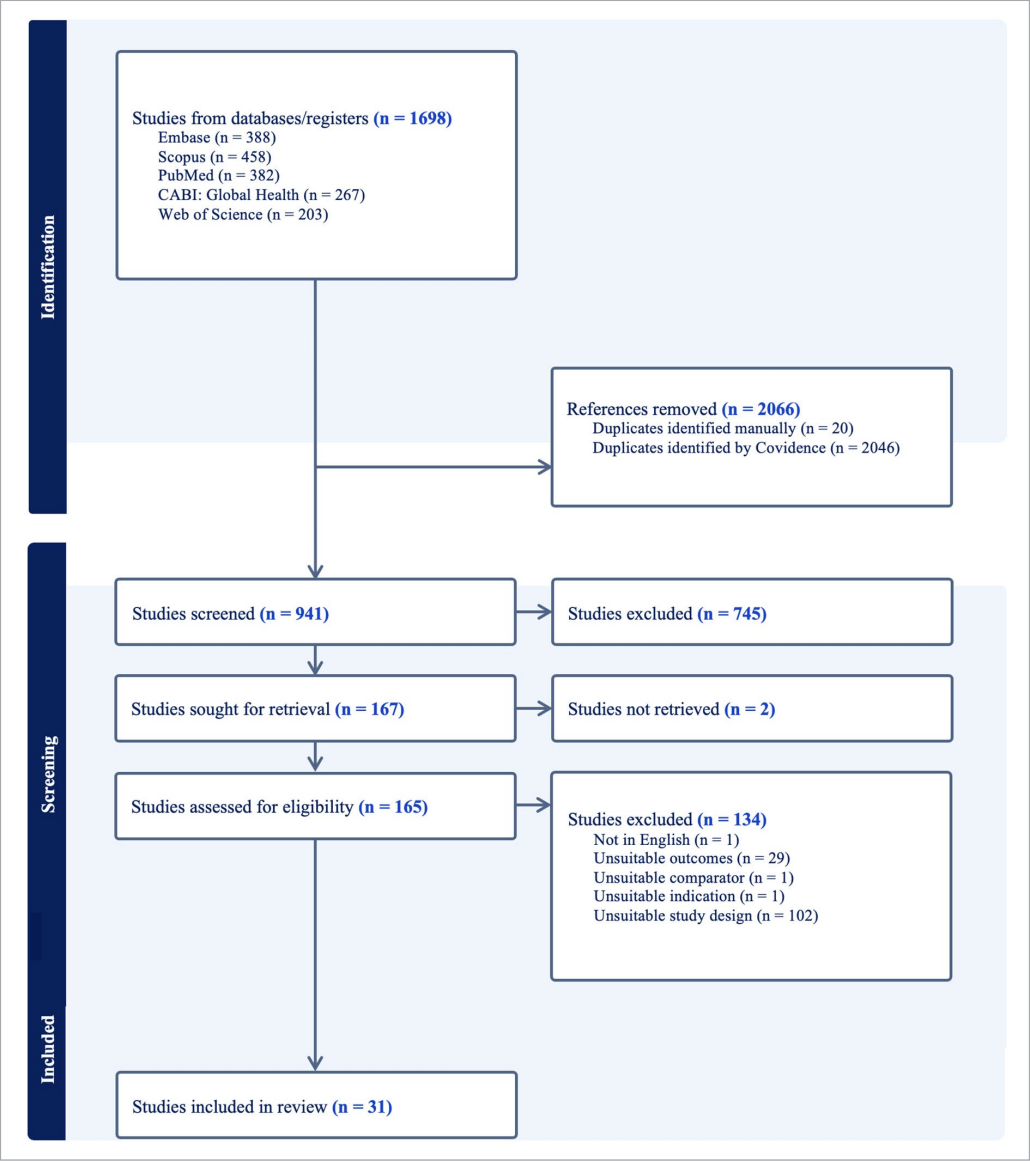
PRISMA Flow diagram depicting study selection process.

The inclusion/exclusion criteria applied to the screening can be found in Table 1. The screening process was conducted in two stages. In the first stage, two authors independently reviewed the titles and abstracts of all identified studies to determine their eligibility based on the predefined inclusion and exclusion criteria. Studies that met the criteria or those with ambiguous relevance were carried forward to the next stage. In the second stage, two senior authors independently reviewed the full texts of the remaining studies to confirm their eligibility for inclusion. Any disagreements at either stage were resolved through discussion and consensus among all four reviewers. The number of articles screened during this process can be found in the Preferred Reporting Items for Systematic Reviews and Meta-Analyses (PRISMA) flowchart in Fig 1.

#### Exclusion criteria

Exclusion criteria were established to ensure that the study maintained focus on traditional and cultural oral hygiene practices while isolating their effects on oral health outcomes. Animal and in-vitro studies as well as single-arm studies without a conventional comparator were excluded. Cancer patients were excluded because cancer and its associated treatments (e.g., chemotherapy, radiotherapy) can strongly influence oral health, introducing confounding factors unrelated to the interventions being studied. Non-oral clinical outcomes and cancer-related outcomes were excluded to narrow the focus to oral health-specific measures, and finally, qualitative research, systematic reviews, commentaries, and case reports were excluded to prioritize high-quality, empirical evidence from randomized controlled trials (RCTs) and quasi-experimental studies, which are better suited for evaluating causal relationships and intervention efficacy. Studies evaluating only conventional methods with no traditional practice arm were also excluded, and outcomes limited to cosmetic effects (e.g., whitening) were not considered unless accompanied by validated plaque/gingival indices.

### Data Extraction and Synthesis

Data on study design, sample size, intervention type, outcomes assessed, and key findings were extracted. Additional variables collected included author, year of publication, country of study, study design, purpose/aim, intervention details, control group details, total sample size, and sample characteristics (e.g., age, sex, population type). The clinical oral health outcomes assessed included plaque accumulation, gingival inflammation, microbial load, and periodontal disease symptoms. Effect measures varied across studies and included mean differences in clinical indices (e.g., plaque index, gingival index, bleeding on probing), percentage reductions in microbial counts (e.g., *S. mutans, A. actinomycetemcomitans*), and statistical significance levels (e.g., p-values, confidence intervals). Two authors independently extracted data from each included study to ensure accuracy and completeness. A second pair of authors validated the extracted data by cross-checking for consistency and resolving discrepancies. Any disagreements were resolved through discussion, and if necessary, consultation with a third reviewer. No automation tools were used in the data extraction process.

Studies meeting the inclusion criteria were categorized based on intervention type (e.g., miswak, oil pulling, herbal dentifrices). Results of individual studies were tabulated to facilitate comparison of extracted data and synthesized findings. A summary table (Table 1) was created to present key study characteristics, including author, year, study design, country, purpose, intervention, control, sample size, participant characteristics, and key findings. This table allowed a comparison of traditional oral hygiene practices and their reported clinical outcomes. Before synthesis, extracted data were reviewed for completeness and consistency.

Due to variability in populations, intervention protocols, and outcome measures, methodological heterogeneity was anticipated. However, no formal heterogeneity analyses (e.g., subgroup analysis or meta-regression) were conducted due to the diversity of study methodologies and outcome reporting. Similarly, no formal sensitivity analyses were performed, as findings were synthesized narratively rather than through quantitative meta-analysis. Instead, robustness was considered qualitatively by identifying consistent patterns, discrepancies, and methodological differences across studies. The potential impact of study quality is discussed in the limitations section.

### Bias and Quality

Risk of bias and study quality were assessed using the Effective Public Health Practice Project (EPHPP) Quality Assessment Tool.^6^ Each study received ratings for selection bias, study design, confounders, blinding, data collection methods, and withdrawals/dropouts. These ratings were summarized in a separate table (Table 3) to provide a transparent evaluation of study quality. One reviewer independently assessed each included study for risk of bias, assigning ratings of strong, moderate, or weak based on predefined EPHPP criteria. Discrepancies in assessments were resolved through discussion with a second reviewer. Studies deemed as weak in terms of quality (as per the EPHPP tool) were noted but still included in narrative synthesis.

**Table 3:** Comparative evaluation of alternative oral hygiene practices based on global ratings

Author, year	Title	Selection bias global rating	Study design global rating	Confounders global rating	Blinding global rating	Data collection methods global rating	Withdrawals and drop-outs global Rating	Final global rating
Miswak
Albabtain et al, 2018	Chemical effects of chewing sticks made of *Salvadora persica*	Moderate	Strong	Weak	Strong	Strong	Strong	Moderate
Al-Otaibi et al, 2004	Subgingival plaque microbiota in Saudi Arabians after use of miswak chewing stick and toothbrush	Weak	Strong	Weak	Moderate	Strong	Weak	Weak
Danielsen et al, 1989	Chewing sticks, toothpaste, and plaque removal	Strong	Strong	Weak	Weak	Strong	Moderate	Weak
Gazi et al, 1992	The immediate- and medium-term effects of meswak on the composition of mixed saliva	Weak	Moderate	Strong	Moderate	Strong	Weak	Weak
Sofrata et al, 2011	Short term clinical effect of active and inactive *Salvadora persica* miswak on dental plaque and gingivitis	Moderate	Strong	Weak	Strong	Strong	Strong	Moderate
Stander and Vaan Wyk 1991	Toothbrushing with the root of *Euclea natalensis*	Moderate	Weak	Weak	Weak	Strong	Not applicable	Weak
Temkar and Menon 2021	Are chewing sticks effective in decreasing oral malodour? A bioanalytical evaluation	Moderate	Strong	Strong	Weak	Strong	Weak	Weak
van Palenstein Helderman et al, 1992	Cleaning effectiveness of chewing sticks among Tanzanian schoolchildren	Strong	Strong	Moderate	Weak	Strong	Weak	Weak
Oil Pulling
Asokan et al, 2008	Effect of oil pulling on *Streptococcus mutans* count in plaque and saliva using Dentocult SM Strip mutans test: a randomized, controlled, triple-blind study	Weak	Strong	Weak	Strong	Strong	Weak	Weak
Kolhe et al, 2019	Oil pulling as an adjunct to improve oral health in orthodontic patients: A clinicomicrobial study	Moderate	Strong	Weak	Weak	Strong	Weak	Weak
Nagilla et al, 2017	Comparative evaluation of antiplaque efficacy of coconut oil pulling and a placebo, among dental college students: A randomized controlled trial	Strong	Strong	Strong	Strong	Moderate	Strong	Strong
Salian et al, 2019	Efficacy of virgin coconut oil and chlorhexidine as an oral antimicrobial: A comparative pilot study	Moderate	Strong	Weak	Strong	Moderate	Weak	Weak
Shetty et al, 2019	Effect of oil pulling on oral health-a microbiological study	Moderate	Strong	Weak	Weak	Weak	Weak	Weak
Sood et al, 2014	Comparative efficacy of oil pulling and chlorhexidine on oral malodor: a randomized controlled trial	Weak	Strong	Strong	Strong	Strong	Strong	Moderate
Herbal toothpastes/dentifrices
Benly, 2015	Comparison of the bacterial level by pre brushing and post brushing using herbal and fluoridated toothpaste	Weak	Strong	Weak	Weak	Strong	Weak	Weak
Devi and Rajasekar, 2022	Effect of herbal and nonherbal dentifrice on gingival health-A clinical study	Moderate	Strong	Weak	Weak	Moderate	Weak	Weak
Howshigan et al, 2015	The effects of an Ayurvedic medicinal toothpaste on clinical, microbiological and oral hygiene parameters in patients with chronic gingivitis: a double-blind, randomised, placebo-controlled, parallel allocation clinical trial	Moderate	Strong	Weak	Strong	Strong	Strong	Moderate
Jayashankar et al, 2011	A randomised double-blind placebo-controlled study on the effects of a herbal toothpaste on gingival bleeding, oral hygiene and microbial variables	Moderate	Strong	Weak	Strong	Strong	Strong	Moderate
Shetty et al, 2017	Comparative evaluation of effect of use of toothbrush with paste and munident on levels of *Streptococcus mutans* and gingival health in children: An in vivo study	Moderate	Strong	Weak	Weak	Strong	Weak	Weak
Herbal mouthwash/oral rinse
Ahmed et al, 2023	Evaluation of the effect of sumac extract and chlorhexidine as mouthwashes on *Streptococcus mutans* in saliva in a group of Egyptian Children	Moderate	Strong	Weak	Weak	Strong	Weak	Weak
Chelli-Chentouf et al, 2012	In vitro and in vivo antimicrobial activity of Algerian Hoggar *Salvadora persica* L. extracts against microbial strains from children’s oral cavity	Moderate	Strong	Weak	Moderate	Strong	Weak	Weak
Dahal et al, 2018	Effectiveness of herbal mouthwash among visually impaired residential school students	Moderate	Strong	Weak	Strong	Strong	Strong	Moderate
Ebrahimian et al, 2019	The effect of Zufa versus chlorhexidine gluconate mouthwashes on oral flora of patients under mechanical ventilation in the intensive care unit: a double-blind, randomized clinical trial	Strong	Strong	Strong	Strong	Strong	Strong	Strong
Hashemi et al, 2019	The efficacy of asafoetida (*Ferula assa-foetida* oleo-gum resin) versus chlorhexidine gluconate mouthwash on dental plaque and gingivitis: A randomized double-blind controlled trial	Moderate	Strong	Moderate	Strong	Strong	Strong	Strong
Mishra et al, 2014	Antimicrobial and plaque inhibitory potential of herbal and probiotic oral rinses in children: a randomized clinical trial	Moderate	Strong	Weak	Strong	Strong	Weak	Weak
Shashikumar et al, 2022	Effect of *Morinda citrifolia* L. mouthwash on periodontal health in type 2 diabetes mellitus patients – a randomized controlled trial	Moderate	Strong	Weak	Strong	Strong	Strong	Moderate
Swastini et al, 2019	The difference between gargling using betel nut seed (*Areca catechu*) extract and chlorhexidine 0.2% solution in chronic gingivitis healing	Moderate	Strong	Weak	Weak	Weak	Weak	Weak
Finger toothbrush / natural toothbrush
Chhaliyil et al, 2020	Impact of different bedtime oral cleaning methods on dental-damaging microbiota levels	Weak	Strong	Weak	Weak	Strong	Weak	Weak
Kulkarni et al, 2023	Baby finger toothbrush as an innovative alternative for finger method of teeth cleaning to improve oral hygiene in rural India	Moderate	Strong	Strong	Moderate	Strong	Weak	Moderate
Parajas 1987	The effectiveness and acceptability of indigenous toothbrush materials among schoolchildren in Aguinaldo, Cavite	Moderate	Weak	Weak	Moderate	Moderate	Weak	Weak
Plant extracts
Patel and Venkatakrishna, 1988	Folklore therapeutic indigenous plants in periodontal disorders in India (review, experimental and clinical approach)	Weak	Moderate	Weak	Weak	Weak	Weak	Weak


No formal certainty assessment was conducted. However, confidence in the body of evidence was considered qualitatively by evaluating study quality, consistency of findings across studies, and methodological limitations. No formal statistical assessment of reporting bias was performed, and this review describes reported outcomes as presented in the included studies without evaluating whether all prespecified outcomes were fully reported. As such, the potential for selective outcome reporting remains a limitation. The influence of risk of bias on study conclusions is discussed in the limitations section.

## RESULTS

### Study Characteristics

Of the 165 full-text articles reviewed for eligibility, 134 studies were excluded. The most common reason for exclusion was unsuitable study design (Fig 1). The 31 studies included in our review spanned 14 different countries, with the highest number of studies conducted in India.

### Intervention Effect

Our systematic review analyzed various traditional oral hygiene practices, including miswak (or chewing sticks), herbal products, oil pulling therapies, and indigenous toothbrush materials. Study sample sizes ranged from small pilot studies to larger trials. Miswak and oral rinses/mouthwashes were the most frequently studied interventions, with evidence suggesting it effectively reduces plaque and improves oral health outcomes, often performing comparably to or exceeding conventional toothbrushes and commercial toothpaste. Oil pulling therapies, primarily using sesame and coconut oils, were associated with moderate antimicrobial effects and reductions in oral malodor. However, these effects were often not statistically significant compared to control treatments such as chlorhexidine. Herbal products, rinses, and indigenous toothbrush materials also demonstrated potential benefits, although evidence was more variable and often population-specific. As stated in the Methods section, no statistical synthesis was conducted due to variability in study methodologies, interventions, and outcome measures. Findings were synthesized narratively, with study-specific effect estimates presented in Table 1, where available, and qualitatively described when not reported.

### Quality/Risk of Bias of Included Studies

While no formal investigations of heterogeneity or sensitivity analyses were performed, variability across studies was examined narratively, and the robustness of findings was considered in relation to study quality and risk of bias, as summarized in Table 3, which presents a comprehensive evaluation of the reviewed studies. Most studies had strong ratings in selection bias and study design, indicating generally well-structured methodologies. However, common weaknesses including lack of confounder adjustment, limitations in blinding procedures, and high withdrawal and dropout rates, likely impacted the reliability of the results. While the results presented in Table 1 suggest potential efficacy for alternative practices, these methodological weaknesses, along with variability in outcome measures, mean that these results should be interpreted cautiously in terms of the effectiveness of alternative oral hygiene practices.

## DISCUSSION

This review addressed how traditional oral hygiene practices compare with conventional ones by looking at numerous studies on traditional oral hygiene practices. The latter remain widespread and culturally significant across multiple regions worldwide. Despite the effectiveness of modern dental care, we observed that traditional practices like miswak use, oil pulling, and herbal mouthwashes may offer promising results in plaque reduction and gingival health, compared to conventional methods. These practices not only provide a cost-effective solution but also align with growing global preferences for sustainable, natural alternatives in healthcare.

Our review highlights the potential efficacy of traditional practices in maintaining oral health, although their outcomes vary depending on the specific methods and contexts. Most studies on miswak (*Salvadora persica*) demonstrated its ability to reduce dental plaque and gingival inflammation, likely due to its unique chemical properties.^44,47^ The reviewed studies suggest that these outcomes may be related to miswak’s antimicrobial compounds and the influence of chewing miswak on the chemical composition of saliva. For example, miswak use was associated with reduced levels of *Aggregatibacter actinomycetemcomitans* in subgingival plaque,^2^ elevated salivary calcium and chloride concentrations, and lower pH and phosphorus levels, which may promote a healthier oral environment.^20^ Although one study reported no statistically significant differences in subgingival microbiota or approximal plaque levels between active miswak (contains bioactive compounds, e.g., antimicrobials) and inactive miswak (lacks bioactive compounds; inactived usually by boiling),^4^ these results may reflect variations in application techniques or differences in the targeted bacterial species. Similarly, a study on the use of *Euclea natalensis* roots, while methodologically weak, found no statistically significant differences in plaque scores, periodontal indices, or DMFT scores between users and non-users. However, the study reported statistically significant tooth discoloration among users, highlighting potential esthetic drawbacks. Miswak’s cultural and historical relevance, combined with its pharmacological benefits, underscores its viability as a primary oral hygiene tool, particularly in communities with limited access to commercial dental products. Miswak’s ecological advantages, such as biodegradability, align with a sustainable approach to oral care, positioning miswak as an effective and environmentally friendly alternative^11^ to conventional oral hygiene methods.

Similarly, oil pulling, especially with sesame and coconut oils, showed modest benefits in reducing microbial load, plaque formation, and gingival index scores, suggesting antimicrobial benefits as well as possible periodontal disease mitigation. Oil pulling has been associated with positive changes in salivary properties, such as buffering capacity and antioxidant levels, comparable to chlorhexidine,^52^ suggesting that oil pulling may be a potentially viable alternative to chlorhexidine in terms of efficacy for individuals seeking a natural, well-tolerated approach to oral hygiene. Results regarding its long-term efficacy and compliance, however, remain inconsistent.^45,60^ Participant acceptance is an important consideration, crucial in the feasibility of oil pulling as a therapy. Kolhe et al^32^ reported that participants found oil pulling more acceptable than chlorhexidine mouthwash due to fewer complaints about taste and discomfort. In contrast, Sood et al^54^ noted that the long duration required for oil pulling was a statistically significant disadvantage for participants, raising concerns about its practicality for sustained use. This time burden reflects protocols that require uninterrupted swishing for several minutes (often 5 to 20), which exceeds the typical 2 to 3 minutes recommended for toothbrushing and may reduce adherence in routine use.

The use of herbal products in toothpastes and mouthwashes is another growing trend, not only due to their antimicrobial properties but also for their alignment with eco-friendly and non-toxic formulations preferred by many users today. Dentifrice formulations containing ingredients such as neem, clove, and pomegranate are shown to possess strong antibacterial and anti-inflammatory properties, comparable to fluoridated products in reducing plaque and supporting gingival health.^31,51^ The studies in this review primarily tested herbal and Ayurvedic formulations incorporating a variety of natural herbs. These studies demonstrated statistically significant reductions in plaque index, bleeding on probing, pocket depth, and salivary anaerobic bacterial counts in participants using herbal toothpaste, with outcomes comparable to those achieved with non-herbal formulations. Only one study found lower bacterial levels in individuals using fluoridated toothpaste compared to herbal or non-fluoridated, non-herbal toothpaste.^8^ It is important to note, however, that these findings were descriptive and that comparisons were not subjected to statistical testing.

Herbal mouthwashes have also shown promising results. Several studies indicated that herbal rinses were as effective as, or in some cases more effective than, chlorhexidine in reducing plaque accumulation, gingival scores, and bacterial counts such as *S. viridans* and *S. mutans*. For example, an asafoetida (*Ferula assa-foetida*) rinse demonstrated greater reductions in plaque index compared to chlorhexidine (mean difference: 1.8 ± 0.6 vs 0.9 ± 0.6; p < 0.0001),^26^ and herbal rinses were comparable to 0.2% chlorhexidine digluconate in clinical performance.^16,36,56^ Both herbal and chlorhexidine mouthwashes statistically significantly reduced plaque and gingival scores more than placebos, with no statistically significant differences in their efficacy on clinical parameters. However, probiotic rinses proved less effective,^36^ and some interventions, such as *M. citrifolia* mouthwash combined with scaling and root planing, did not yield additional benefits over scaling and root planing alone.^48^

Our findings support the idea that herbal toothpaste and mouthwashes may be just as effective as conventional products for improving gingival health, providing consumers with a natural alternative without compromising efficacy. Additionally, the low toxicity and non-irritating nature of herbal mouthwashes make them viable for long-term use, presenting a favorable alternative to synthetic options. While not tested in any of our reviewed studies, Ayurvedic powders, utilizing natural ingredients like amla and tulsi, provide additional preventive and therapeutic benefits, catering to the demand for more natural, less processed products in oral hygiene.^18^ The integration of herbal ingredients into conventional oral care formulations could enhance consumer choice while promoting safer, environmentally sound hygiene practices.^20^

Finger brushing and the use of indigenous toothbrush materials, such as coconut husks, betelnut, and guava twigs, emerged as potentially effective traditional practices for oral hygiene, particularly in resource-limited settings. Integrating these findings into the broader discussion of traditional oral hygiene practices reinforces the idea that simple, culturally embedded methods can achieve comparable outcomes to modern interventions. Finger brushing with herbal or charcoal formulations and indigenous toothbrush materials not only provide accessible, affordable options but also align with sustainability and waste reduction goals, given their natural origins and biodegradability. Future research should explore the long-term impact of these practices on oral microbiota composition and clinical outcomes, as well as their acceptability across diverse populations. Such studies could further inform public health strategies aimed at expanding access to effective and culturally appropriate oral hygiene solutions.

As can be seen in Table 3, the quality of evidence across most of the studies reviewed was generally low. This highlights the need for further research to evaluate the long-term benefits, limitations, and acceptability of traditional oral hygiene practices, particularly in diverse populations and resource-limited settings. Higher-quality studies are essential to establish the efficacy, impact on oral microbiota, and long-term acceptability of these practices, particularly in comparison to conventional oral care methods.

This review is limited by methodological heterogeneity across included studies, with variations in populations, intervention protocols, and outcome measures. While findings were synthesized narratively, the lack of formal heterogeneity analysis (e.g., subgroup analysis, meta-regression) limits the ability to quantify variability. Similarly, no formal sensitivity analyses were conducted, making it difficult to assess the robustness of findings across different study characteristics.

Study quality varied, with some studies lacking blinding or adequate confounder control, which may introduce bias in reported outcomes. Although studies rated as “weak” quality (per EPHPP) were included, their methodological limitations should be considered when interpreting results. Additionally, this review did not conduct a formal certainty assessment, which limits the ability to categorize the strength of evidence systematically.

Most of the studies lacked standardized protocols, limiting direct comparisons with conventional methods. Furthermore, while many studies demonstrate the short-term benefits of these traditional practices, evidence on their long-term effects, especially on systemic health, remains sparse. Future research should prioritize longitudinal studies that assess the durability of these benefits and investigate potential adverse effects that might arise from prolonged use of certain natural products. Additionally, studies should evaluate the most effective education programs to maximize the benefits and proper use of traditional oral hygiene products. Rigorous, multicentric, double-blinded trials across diverse populations would strengthen the evidence base, ensuring that these practices are evaluated with scientific precision and cultural sensitivity.

Finally, limiting this review to English-only publications is an additional limitation, as it may have excluded relevant studies published in other languages, particularly those from regions where traditional oral hygiene practices are prevalent.^42,46^ Addressing this language bias in future reviews would provide a more comprehensive understanding of the global evidence base.

## CONCLUSION

Our review highlights the potential of traditional oral hygiene methods in addressing health disparities, particularly in underserved communities. In line with our stated question, these traditional practices can achieve short-term plaque/gingivitis reductions comparable to conventional care when correctly used, albeit with heterogeneous methods and limited long-term data. These culturally informed practices offer accessible, affordable solutions tailored to local needs and could play a key role in improving oral health standards.^35^ Integrating traditional knowledge with modern scientific approaches can enhance global oral health, but continued research and education are crucial to ensure these practices are safe, effective, and culturally sensitive. This balanced approach not only provides more choices for consumers but also supports better access to oral care across diverse populations.

## ACKNOWLEDGMENTS

This review did not receive any financial support. However, access to Covidence for screening and data extraction was provided by Emory University, which we acknowledge as a form of non-financial support.

## Appendix

**Table 2:** Summary of studies evaluating the efficacy of traditional oral hygiene practices

Author, year	Study design	Country	Purpose/Aim	Intervention	Control	Total Sample Size	Sample Characteristics	Findings
Miswak
Albabtain et al, 2018	Randomized controlled trial	Sweden	Investigate chemical effects of *Salvadora persica* on gingival inflammation and subgingival microflora	Active miswak (*Salvadora persica*)	Inactive miswak	28	Adults with more than 20 teeth, ≥1 pathological gingival pocket	No statistically significant differences in clinical variable (gingival inflammation, bleeding on probing and plaque index) at baseline and follow-up between active and inactive miswak users. No statistically significant differences in oral microbial diversity after using active and inactive miswak.
Al-Otaibi et al, 2004	Randomized controlled trial	Saudi Arabia	Compare effects of miswak vs toothbrush on subgingival plaque microflora	Miswak (*Salvadora persica*)	Toothbrush	15	Healthy adults with ≥24 teeth, non-smokers	After the miswak period, subjects had statistically significantly lower levels of* A. actinomycetemcomitans* in subgingival plaque. No such reduction was found after the toothbrushing period. There were no statistically significant changes in the abundance of the remaining 11 bacterial species, regardless of miswak or toothbrushing periods.
Danielsen et al, 1989	Randomized controlled trial	Kenya	Assess efficacy of brushing with chewing sticks vs toothpaste for plaque removal	Chewing stick (maswak) and toothpaste	Chewing stick (maswak) only	70	Habitual user of chewing stick	Brushing with a chewing stick for 5 min resulted in a small net reduction of the proportion of plaque deposit sites per child, however, toothpaste resulted in no additional effect.
Gazi et al, 1992	Randomized controlled tria	Saudi Arabia	Investigate effects of meswak on saliva composition and oral health	Meswak (*Salvadora persica*)	Pyrogen-free rubber, toothbrush	20	Healthy adults, high oral hygiene standard, never used meswak for cleaning teeth.	Salivary calcium and chloride concentrations were statistically significantly higher, whereas the pH and phosphorus were statistically significantly lower, after chewing with meswak compared to rubber. Calcium and chloride values were similar to those of controls after 4 h Scores for plaque and gingival indices were statistically significantly lower following use of meswak compared to the conventional toothbrush.
Sofrata et al, 2011	Randomized controlled trial	Saudi Arabia	Evaluate effect of active and inactive miswak on dental plaque and gingival inflammation	Active miswak (*Salvadora persica*)	Inactive miswak	68	18 years of age, at least 24 teeth	Active miswak statistically significantly reduced supragingival plaque (p = 0.007); however, no differences were observed in approximal plaque levels or subgingival microbiota between the active and inactive miswak groups.
Stander and Vaan Wyk, 1991	Non-randomized experimental study	South Africa	Assess clinical effect of *Euclea natalensis* roots on oral health	*Euclea natalensis* roots	Non-user of *Euclea* roots	90	Zanzabari women	Discoloration of teeth was observed in users. No statistically significant differences in the plaque score, periodontal index and DMFT score between users and non-users.
Temkar and Menon, 2021	Randomized controlled trial (three-way cross-over design)	India	Evaluate efficacy of chewing sticks (neem and miswak) in decreasing oral malodor	Neem (*Azadirachta indica*), Miswak (*Salvadora persica*)	Close Up® toothpaste	6	Healthy males (18-65 years), ≥26 natural permanent teeth, no systemic disorders	While all treatments were effective in decreasing volatile sulfur (VSCs) and other malodour-causing compounds in mouth air samples, both chewing sticks (neem and miswak) yielded a greater decrease in VSCs compared to the toothpaste.
van Palenstein Helderman et al, 1992	Non-randomized controlled trial	Tanzania	Evaluate whether an oral health education program could improve oral health in school program	Oral health education program	Toothbrush	124	School children, habitual miswak users	Both chewing stick and toothbrush users in the experimental schools statistically significantly reduced plaque and gingival bleeding scores No substantial changes in oral hygiene occurred in the control group.
Oil pulling
Asokan et al, 2008	Randomized controlled trial	India	Evaluate effect of sesame oil pulling on *Streptococcus mutans* count in plaque and saliva	Sesame oil	Chlorhexidine mouthwash	20	Adolescent boys aged 16-18, DMF score of 1-2	Statistically significant reduction in *S. mutans* in the plaque of the oil pulling group after 1 (p = 0.001) and 2 weeks (p = 0.008), however, not in the saliva. Statistically significant reduction in both saliva and plaque *S. mutans* counts at all time points in control (chlorhexidine group)
Kolhe et al, 2019	Randomized controlled trial	India	Evaluate effect of oil pulling with sesame oil in orthodontic patients	Sesame oil	Chlorhexidine gluconate mouthwash	20	Individuals undergoing orthodontic treatment	Oil pulling therapy showed a reduction in total colony counts of aerobic microorganisms. Oil pulling more acceptable to patients compared to the control mouthwash, with fewer complaints of discomfort or taste alteration and higher compliance
Nagilla et al, 2017	Randomized controlled trial	India	Compare antiplaque efficacy of coconut oil pulling with placebo	Coconut oil	Placebo (mineral water)	40	Healthy individuals aged 18-22, ≥20 natural teeth	Mean plaque score was statistically significantly lower among oil pulling group on seventh day in comparison with the control group (p < 0.001) Study group had a statistically significantly higher mean plaque reduction on the 7th day compared to control group (p < 0.001)
Salian et al, 2019	Non-randomized controlled trial (double blind)	India	Evaluate efficacy of oil pulling therapy using virgin coconut oil in reducing *S. mutans*, plaque, and gingival indices	Virgin coconut oil	Chlorhexidine	40	Adults aged 18+ with carious teeth and moderate-severe gingival inflammation	No statistically significant differences in gingival and plaque index and *S. mutans* count after 3 weeks oil pulling.
Shetty et al, 2019	Randomized controlled trial	India	Evaluate effect of oil pulling with coconut oil compared to chlorhexidine	Coconut oil	Chlorhexidine mouthwash	20	Students aged 19-23 years	Oil pulling therapy and use of chlorhexidine showed reduction in plaque formation and number of colony forming units, however, differences were not statistically significant.
Sood et al, 2014	Randomized controlled trial	India	Compare efficacy of sesame oil pulling vs chlorhexidine in reducing oral malodor and microbes	Sesame oil Chlorhexidine	Placebo	60	Adults with ≥24 permanent teeth, gingival probing depth <3mm	Statistically significant reduction (p < 0.05) in mean scores of all parameters within sesame oil and chlorhexidine group Post intervention, both sesame oil and chlorhexidine groups demonstrated significantly lower (p = 0.000) mean organoleptic scores and bacterial colony counts (p = 0.000) compared to placebo group No statistically significant differences (p < 0.05) were observed between sesame oil and chlorhexidine groups.
Herbal toothpastes/dentifrices
Benly, 2015	Non-randomized experimental study	India	Compare bacterial levels on teeth before and after brushing with herbal vs fluoridated toothpaste	Herbal toothpaste with *Terminalia chebula *	Fluoridated and non-fluoridated non-herbal toothpaste	15	Healthy individuals with no dental caries	Descriptive findings show that bacterial levels were lower in individuals using fluoridated toothpaste compared to those using herbal or non-fluoridated, non-herbal toothpaste.
Devi and Rajasekar, 2022	Randomized controlled trial	India	Evaluate impact of herbal and non-herbal toothpaste on gingival health	Herbal toothpaste (Colgate Herbal)	Non-herbal toothpaste (Colgate)	100	Patients with generalized gingivitis, minimum 20 teeth	Both herbal and non-herbal toothpaste groups demonstrated reduced plaque and gingival index values with no statistically significant difference between groups suggesting that herbal dentifrices are just as effective as nonherbal dentifrices at reducing plaque and improving gingival health
Howshigan et al, 2015	Randomized controlled trial	Sri Lanka	Study efficacy of Ayurvedic toothpaste containing various herbs for chronic gingivitis	Sudantha® toothpaste	Placebo toothpaste	80	Patients with chronic gingivitis	Statistically significant reductions of plaque, bleeding on probing, pocket depth (p < 0.0001) and total salivary anaerobic bacteria counts (p < 0.05) in patients using Ayurvedic toothpaste at all prescribed visits compared to placebo
Jayashankar et al, 2011	Randomized controlled trial	Sri Lanka	Investigate oral hygiene benefits of herbal toothpaste	Herbal toothpaste (Sudantha®)	Placebo toothpaste	60	Healthy dental students aged 18-35	Plaque index, bleeding on probing and salivary anaerobic bacterial counts decreased statistically significantly in participants using herbal toothpaste at 4, 8, and 12 weeks compared to baseline measurement. In the placebo group, measurements of all variables at all time points were not statistically significantly different when compared to baseline measurements
Shetty et al, 2017	Non-randomized controlled study	India	Compare antibacterial efficacy of Munident (Ayurvedic dentifrice) vs commercially available standard toothpaste	Munident herbal dentifrice	Standard toothpaste	40	Healthy children (9-12 years), DMFT score ≥3	Both Munident (herbal) dentifrice and standard toothpaste demonstrated a statistically significant reduction in the levels of *S. mutans* and gingival bleeding index (GBI). There was no evidence, however, to indicate that Munident performed better than standard toothpaste in terms of bacterial count or GBI.
Herbal mouthwash/oral rinse
Ahmed et al, 2023	Randomized controlled trial	Egypt	Evaluate antibacterial effect of ginger aqueous extract vs chlorhexidine on *Streptococcus mutans*	Ginger aqueous extract	Chlorhexidine mouthwash	60	Children with low caries index, no history of dental prophylaxis for at least 3 months, no permanent or removable orthodontic appliances	*S. mutans* numbers decreased significantly across all groups, with chlorhexidine mouthwash having the greatest mean value of percentage change (73.84 ± 4.348 %) There was no statistical difference between the 20% ginger extract group and the chlorhexidine.
Chelli-Chentouf et al, 2012	Randomized controlled trial	Algeria	Assess antimicrobial activity of miswak extract on oral bacteria in children	Miswak (*Salvadora persica*) extract	A placebo mouthwash made of sterile demineralized water	40	Children aged 6-12, no gum disease, no recent antibiotic use	In vitro: Miswak extract showed greater inhibition of Gram-negative bacterial growth in dental plaque compared to Gram-positive bacteria. In vivo: A statistically significant reduction in bacteria of the oral cavity using miswak mouthwash as compared to placebo.
Dahal et al, 2018	Randomized controlled trial	Nepal	Assess effectiveness of herbal mouthwash vs chlorhexidine in reduction of plaque and gingivitis in visually impaired children	Herbal mouthwash (Hiora)	Chlorhexidine mouthwash, placebo	82	Visually impaired students (6-20 years) with gingivitis	Both herbal mouthwash and chlorhexidine statistically significantly reduced plaque and gingival scores more than the placebo. There was no statistically significant difference between herbal mouthwash and chlorhexidine performance on these clinical parameters.
Ebrahimian et al, 2019	Randomized controlled trial	Iran	Compare effect of Zufa and chlorhexidine mouthwash on oral flora in ICU patients under mechanical ventilation	Zufa (*Hyssopus angustifolius* L) mouthwash	Chlorhexidine gluconate, normal saline	90	Patients under mechanical ventilation, Glasgow Coma Scale <8, no diabetes or oral cavity damage, receiving no food by mouth, no seizures, and no consumption of immuno-suppressive drugs	Statistically significant improvement in the oral health status of the subjects in the Zufa, chlorhexidine gluconate, and normal saline groups on first, second and third day of intervention as assessed by Beck oral assessment scale (BOAS).
Hashemi et al, 2019	Randomized controlled trial	Iran	Investigate efficacy of Asafoetida vs chlorhexidine mouthwash	*Ferula assa-foetida* mouthwash	Chlorhexidine (CHG) mouthwash	126	Adults (18-35 years)	After the intervention period, both asafoetida and CHG groups showed significant improvements in modified gingival index and plaque index (PI) scores Asafoetida group demonstrated greater reductions compared to the CHG group (p < 0.0001), with a larger improvement in PI mean difference (1.8 ± 0.6 vs 0.9 ± 0.6)
Mishra et al, 2014	Randomized controlled trial	India	Evaluate antimicrobial and plaque inhibitory potential of herbal and probiotic rinses against *Streptococcus viridans *	Probiotic mint tablet, Herbal oral rinse	0.2% chlorhexidine digluconate oral rinse	60	Children (6-14 years) with carious teeth, plaque index ≥ 0.9, no history of antimicrobial agent or drug use for past 3 months	Herbal rinse proved equally effective as 0.2% chlorhexidine digluconate in reducing S. viridans counts and plaque accumulation after 1 week of intervention Probiotic rinse was least effective.
Shashikumar et al, 2022	Randomized controlled trial	India	Evaluate effects of *Morinda citrifolia* mouthwash on periodontal outcomes in patients with diabetes and chronic periodontitis	Morinda citrifolia mouthwash + scaling and root planing	Scaling and root planing only	96	Adults (30-60 years) with chronic periodontitis and Type 2 diabetes	Daily rinsing with *M. citrifolia* mouthwash with scaling and root planing treatment did not show any added benefits in terms of clinical periodontal parameters or salivary total antioxidant capacity compared to scaling and root planing alone.
Swastini et al, 2019	Randomized controlled trial	Indonesia	Compare the effectiveness of areca seed extract and chlorhexidine 0.2% in promoting the healing of chronic gingivitis	Areca nut extract	Chlorhexidine 0.2% solution	30	Junior high students diagnosed with grade 2 gingivitis	Mean gingival index decreased statistically significantly after gargling with areca nut seed extract (*Areca catechu*) and vhlorhexidine 0.2% solution. There was no statiscially significant difference in the impact of areca nut seed extract (*Areca catechu*) or chlorhexidine 0.2% solution on gingival index scores.
Finger toothbrush / natural toothbrush
Chhaliyil et al, 2020	Randomized controlled trial	USA	Evaluate efficiency of three oral cleaning methods to remove harmful oral microbiota	GIFT method (cleaning with finger) GIFT method + charcoal Tooth brushing with fluoride toothpaste	No cleaning	58	Healthy children (10-12 years)	All three interventions statistically significant decreased quantity of *Aggregatibacter actinomycetemcomitans* (p < 0.006) GIFT and GIFT + charcoal decreased *A. actinomycetemcomitans* compared to the toothbrushing with fluoride (p < 0.005, p < 0.04).
Kulkarni et al, 2023	Non-randomized experimental study	India	Compare efficacy of baby finger toothbrush vs finger cleaning	Baby finger toothbrush	Finger cleaning	60	Adults (18-75 years) cleaning teeth with fingers	Oral hygiene status scores were statistically significantly higher in the participants cleaning teeth with their fingers compared to the participants using baby finger toothbrush after 15 days.
Parajas, 1987	Quasi-experimental study	Philippines	Test effectiveness and acceptability of indigenous toothbrush materials	Toothbrushes made from coconut husk material, guava twig and betelnut husk		92	Grade IV pupils of two elementary schools	All indigenous toothbrush materials statistically significantly improved effectiveness of hygiene practices, with statistically significant improvements before and after use, and no statistically significant difference between toothbrushes. The majority of students (60%) preferred coconut husk toothbrushes followed by betelnut (20%) and guava twig (20%)
Plant extracts
Patel and Venkatakrishna-Bhatt, 1988	Quasi-experimental study	India	Evaluate efficacy of medicinal plant leaf extracts as antiplque agent	Neem, Mango, Ocimum, Teadust, Curry leaf extracts		50	Adults aged 20-50 with chronic periodontitis	After three weeks of treatment with the leaf extract (250 mg applied twice daily), 40 patients showed statistically significant improvement in symptoms (bleeding and purulent discharge) and reduction in plaque index scores and reduction in oral microbes with no side effects reported.


**Table A1:** Search strategy and database queries

Database	Date of Search	Search #	Search Strategy	# Results Retrieved
PubMed	1 April 2024	1	(oral[ti] OR dental[ti] OR dentist*[ti] OR tooth*[ti] OR teeth[ti] OR Periodon*[ti] OR “Oral Hygiene”[Mesh] OR “Oral Health”[Mesh])	382
2	(Behavior*[tw] OR Behaviour*[tw] OR Brush*[tw] OR Care[tw] OR Clean*[tw] OR health[tw] OR Hygien*[tw] OR Practice*[tw] OR “Health Behavior”[Mesh] OR “Toothbrushing”[Mesh])
3	(Aborigin*[tw] OR ethno*[tw] OR Folk[tw] OR Indigenous[tw] OR “Non-western”[tw] OR rural[tw] OR tribe*[tw] OR tribal[tw] OR traditional[tw] OR “Pharmacognosy”[Mesh] OR “Medicine, Traditional”[Mesh])
4	(Ash[tw] OR brick[tw] OR Charcoal[tw] OR Herbal[tw] OR mud[tw] OR powder*[tw] OR bark*[tw] OR branch[tw] OR branches[tw] OR twig*[tw] OR stick[tw] OR sticks[tw] OR sprig*[tw] OR Miswak[tw] OR “Salvadora Persica”[tw] OR mastic[tw] OR “Oil pull*”[tw] OR finger*[tw] OR “Plant Extracts”[Mesh] OR “Plants, Medicinal”[Mesh])
5	#1 AND #2 AND #3 AND #4 AND English[lang] AND “journal article”[Publication Type] NOT (“animals”[MeSH Terms] NOT “humans”[MeSH Terms])
Embase	1 April 2024	1	(oral:ti OR dental:ti OR dentist*:ti OR tooth*:ti OR teeth:ti OR Periodon*:ti OR ‘mouth hygiene’/exp)	388
2	(Behavior*:ti,ab,kw OR Behaviour*:ti,ab,kw OR Brush*:ti,ab,kw OR Care:ti,ab,kw OR Clean*:ti,ab,kw OR health:ti,ab,kw OR Hygien*:ti,ab,kw OR Practice*:ti,ab,kw OR ‘health behavior’/exp OR ‘tooth brushing’/exp)
3	(Aborigin*:ti,ab,kw OR ethno*:ti,ab,kw OR Folk:ti,ab,kw OR Indigenous:ti,ab,kw OR “Non-western”:ti,ab,kw OR rural:ti,ab,kw OR tribe*:ti,ab,kw OR tribal:ti,ab,kw OR traditional:ti,ab,kw OR ‘pharmacognosy’/exp OR ‘traditional medicine’/exp)
4	(Ash:ti,ab,kw OR brick:ti,ab,kw OR Charcoal:ti,ab,kw OR Herbal:ti,ab,kw OR mud:ti,ab,kw OR powder*:ti,ab,kw OR bark*:ti,ab,kw OR branch:ti,ab,kw OR branches:ti,ab,kw OR twig*:ti,ab,kw OR stick:ti,ab,kw OR sticks:ti,ab,kw OR sprig*:ti,ab,kw OR Miswak:ti,ab,kw OR “Salvadora Persica”:ti,ab,kw OR mastic:ti,ab,kw OR “Oil pull*”:ti,ab,kw OR finger*:ti,ab,kw OR ‘plant extract’/exp OR ‘medicinal plant’/exp)
5	#1 AND #2 AND #3 AND #4 AND [english]/lim AND ([article]/lim OR [article in press]/lim) NOT ([animals]/lim NOT [humans]/lim)
Global Health	2 April 2024	1	Title:(oral OR dental OR dentist* OR tooth* OR teeth OR Periodon*)	267
2	Title:(Behavior* OR Behaviour* OR Brush* OR Care OR Clean* OR health OR Hygien* OR Practice*) OR Ab:(Behavior* OR Behaviour* OR Brush* OR Care OR Clean* OR health OR Hygien* OR Practice*)
3	Title:(Aborigin* OR ethno* OR Folk OR Indigenous OR “Non-western” OR rural OR tribe* OR tribal OR traditional) OR Ab:(Aborigin* OR ethno* OR Folk OR Indigenous OR “Non-western” OR rural OR tribe* OR tribal OR traditional)
4	Title:(Ash OR brick OR Charcoal OR Herbal OR mud OR powder* OR bark* OR branch OR branches OR twig* OR stick OR sticks OR sprig* OR Miswak OR “Salvadora Persica” OR mastic OR “Oil pull*” OR finger*) OR Ab:(Ash OR brick OR Charcoal OR Herbal OR mud OR powder* OR bark* OR branch OR branches OR twig* OR stick OR sticks OR sprig* OR Miswak OR “Salvadora Persica” OR mastic OR “Oil pull*” OR finger*)
Limits	Item Type = Journal Article
Scopus	3 April 2024	1	TITLE (oral OR dental OR dentist* OR tooth* OR teeth OR Periodon*)	458
2	TITLE-ABS-KEY (Behavior* OR Behaviour* OR Brush* OR Care OR Clean* OR health OR Hygien* OR Practice*)
3	TITLE-ABS-KEY (Aborigin* OR ethno* OR Folk OR Indigenous OR “Non-western” OR rural OR tribe* OR tribal OR traditional)
4	TITLE-ABS-KEY (Ash OR brick OR Charcoal OR Herbal OR mud OR powder* OR bark* OR branch OR branches OR twig* OR stick OR sticks OR sprig* OR Miswak OR “Salvadora Persica” OR mastic OR “Oil pull*” OR finger*)
5	LANGUAGE(english) AND DOCTYPE(ar)
Web of Science	3 April 2024	1	TI=(oral OR dental OR dentist* OR tooth* OR teeth OR Periodon*)	203
2	TS=(Behavior* OR Behaviour* OR Brush* OR Care OR Clean* OR health OR Hygien* OR Practice*)
3	TS= (Aborigin* OR ethno* OR Folk OR Indigenous OR “Non-western” OR rural OR tribe* OR tribal OR traditional)
4	TS=(Ash OR brick OR Charcoal OR Herbal OR mud OR powder* OR bark* OR branch OR branches OR twig* OR stick OR sticks OR sprig* OR Miswak OR “Salvadora Persica” OR mastic OR “Oil pull*” OR finger*)
5	#1 AND #2 AND #3 AND #4 AND LA=(English) AND DT=(Article)

